# Evaluating the impact of using a wound‐specific oral nutritional supplement to support wound healing in a rehabilitation setting

**DOI:** 10.1111/iwj.13849

**Published:** 2022-06-09

**Authors:** Rya K. Clark, Argyrios Stampas, Kirk W. Kerr, Jeffrey L. Nelson, Suela Sulo, Luis Leon‐Novelo, Esther Ngan, Dehuti Pandya

**Affiliations:** ^1^ Clinical Nutrition TIRR Memorial Hermann Hospital Houston Texas USA; ^2^ Spinal Cord Injury Medicine Research TIRR Memorial Hermann Hospital Houston Texas USA; ^3^ Scientific and Medical Affairs Abbott Laboratories Columbus Ohio USA; ^4^ School of Public Health University of Texas Health Science Center at Houston Houston Texas USA; ^5^ Department of Pharmacy TIRR Memorial Hermann Hospital Houston Texas USA

**Keywords:** nutritional support, wound healing, wounds and injuries

## Abstract

Chronic wounds adversely affect patient quality of life, increase the risk of mortality, and impose high costs on healthcare systems. Since protein‐energy malnutrition or specific nutrient deficiencies can delay wound healing, nutritionally focused care is a key strategy to help prevent or treat the occurrence of non‐healing wounds. The objective of our study of inpatients in a rehabilitation hospital was to quantify the effect of daily wound‐specific oral nutritional supplementation (WS‐ONS) on healing chronic wounds. Using electronic medical records, we conducted a retrospective analysis of patients with chronic wounds. We identified records for (a) a treatment group who received standard wound care + usual hospital diet + daily WS‐ONS for ≥14 days, and (b) a control group who received standard wound care + a usual hospital diet. We collected data for demographics, nutritional status, and wound‐relevant health characteristics. We examined weekly measurements of wound number and sizes (surface area for superficial wounds or volume for non‐superficial wounds). There were 341 patients identified, 114 with 322 wounds in the treatment group and 227 patients with 420 wounds in the control group. We found that rehabilitation inpatients who were given nutritional support had larger wounds and lower functional independence on admission. At discharge, wound area reduction (percent) was nearly two‐fold better in patients who were given daily WS‐ONS + usual hospital diet compared to those who consumed usual diet only (61.1% vs 34.5%). Overall, weekly wound improvement (lowered wound area or wound volume) was more likely in the WS‐ONS group than in the Control group, particularly from the start of care to week 2. Inpatients with largest wounds and lowest functional independence on admission were most likely to be given WS‐ONS, an indication that caregivers recognised the need for supplementation. Week‐to‐week improvement in wound size was more likely in patients who received WS‐ONS than in those who did not. Specifically, wound areas and wound volumes were significantly lower at discharge among patients who were given specialised nutritional support. More research in this field is needed to improve care and reduce healthcare costs.

## 1 INTRODUCTION

Wounds adversely affect patient quality of life^1^ and survival,^2^ and markedly increase healthcare needs and costs of care.^3^, ^4^ While hard‐to‐heal wounds can occur in the general population, some people are particularly vulnerable, such as those who are older,^5^ have acute or chronic disease conditions,^5^ are experiencing disability with immobility,^6^, ^7^ or are hospitalised or staying at rehabilitation or nursing care facility.^6^ Common hard‐to‐heal wounds include arterial and venous leg ulcers, diabetic foot ulcers, pressure injuries, skin infections, and surgical wounds.^8^ Hard‐to‐heal wounds are also called chronic wounds, as they fail to proceed through the normal phases of wound healing (haemostasis, inflammatory, proliferative, and maturation) in an orderly and timely manner—often stalling in the inflammatory phase.^9^

Poor nutritional status and inadequate wound healing are inextricably linked. Malnourished patients are vulnerable to pressure injuries and complicating infections and may experience delayed healing of surgical incisions or traumatic injuries.^10^, ^11^, ^12^ Research has shown that specialised nutritional supplementation, in addition to standard wound care, can effectively improve the healing of wounds such as diabetic foot ulcers^13^, ^14^ and pressure injuries.^11^, ^15^, ^16^ Nutrients recognised as crucial to wound healing include adequate energy by intake of carbohydrates and fats, sufficient protein to promote healing processes, and certain conditionally essential amino acids such as arginine and glutamine.^17^ In addition to these amino acids, a metabolite of the amino acid leucine (ie, beta‐hydroxy‐beta‐methylbutyrate, HMB) has been shown to stimulate protein synthesis pathways and decrease protein degradation,^18^ in turn supporting wound healing processes, promoting the healing of burns^19^ and diabetic foot ulcers.^13^ Minerals such as zinc, selenium, and iron are necessary for optimal wound healing by affecting enzyme function.^17^ Deficiencies in key vitamins such as vitamin A (retinoic acid), vitamin C (ascorbic acid), and vitamin D are also implicated in prolonging the wound‐healing process.^17^

In addition to the pain and functional impairment experienced by patients with non‐healing wounds, there are also excessive costs associated with the high use of healthcare resources. Based on United States (US) Medicare records from 2014, wounds impacted nearly 15% of Medicare beneficiaries (mostly adults ≥65 years old), with estimates of associated healthcare costs ranging from $28 billion to $98.6 billion when including wounds secondary to other health conditions.^4^ In US hospital‐based outpatient services, the mean cost‐to‐heal per wound was $3927 for patients with a mean age of 61.7 years.^3^ A recent study of the economic burden of wounds in the United Kingdom (UK) estimated that only 43% of chronic wounds healed during a 1‐year study period; based on these findings, the researchers estimated the cost of managing chronic wounds at £3.0 billion per year.^20^ Altogether, interventions that help prevent or treat pressure injuries and other chronic wounds are expected to decrease costs for wound care and other healthcare services while increasing the quality of life for affected individuals.^21^

The objective of our present study of rehabilitation hospital inpatients was to quantify the effects of daily wound‐specific oral nutritional supplementation (WS‐ONS) on healing wounds.

## 2 METHODS

### 2.1 Study participants and ethical approval

The Institute for Rehabilitation and Research (TIRR) includes the 134‐bed Memorial Hermann Hospital and Research Center in Houston, Texas, USA. Inpatients who had existing wounds and were treated at TIRR between December 1, 2017 and December 31, 2019 were identified in the Electronic Medical Record (EMR) system. For inclusion in the analysis, inpatients had at least one measurable wound and had at least two Wound Care Nurse (WCN) visits for wound care.

The study protocol was reviewed and approved by the Institutional Review Board (IRB) of The University of Texas Health Science Center, Houston, TX.

### 2.2 Study design

The study was an EMR‐based retrospective analysis of patients with existing wounds. We identified records for (a) a Treatment group who received standard wound care + usual hospital diet + daily WS‐ONS (Juven, Abbott Nutrition, Columbus, OH), and (b) a Control group who received usual wound care + a usual hospital diet. We also identified subgroups of patients who spent ≥14 days as inpatients of the rehabilitation hospital and received standard wound care and diet +14 or more days WS‐ONS or standard wound care and diet.

**FIGURE 1:**
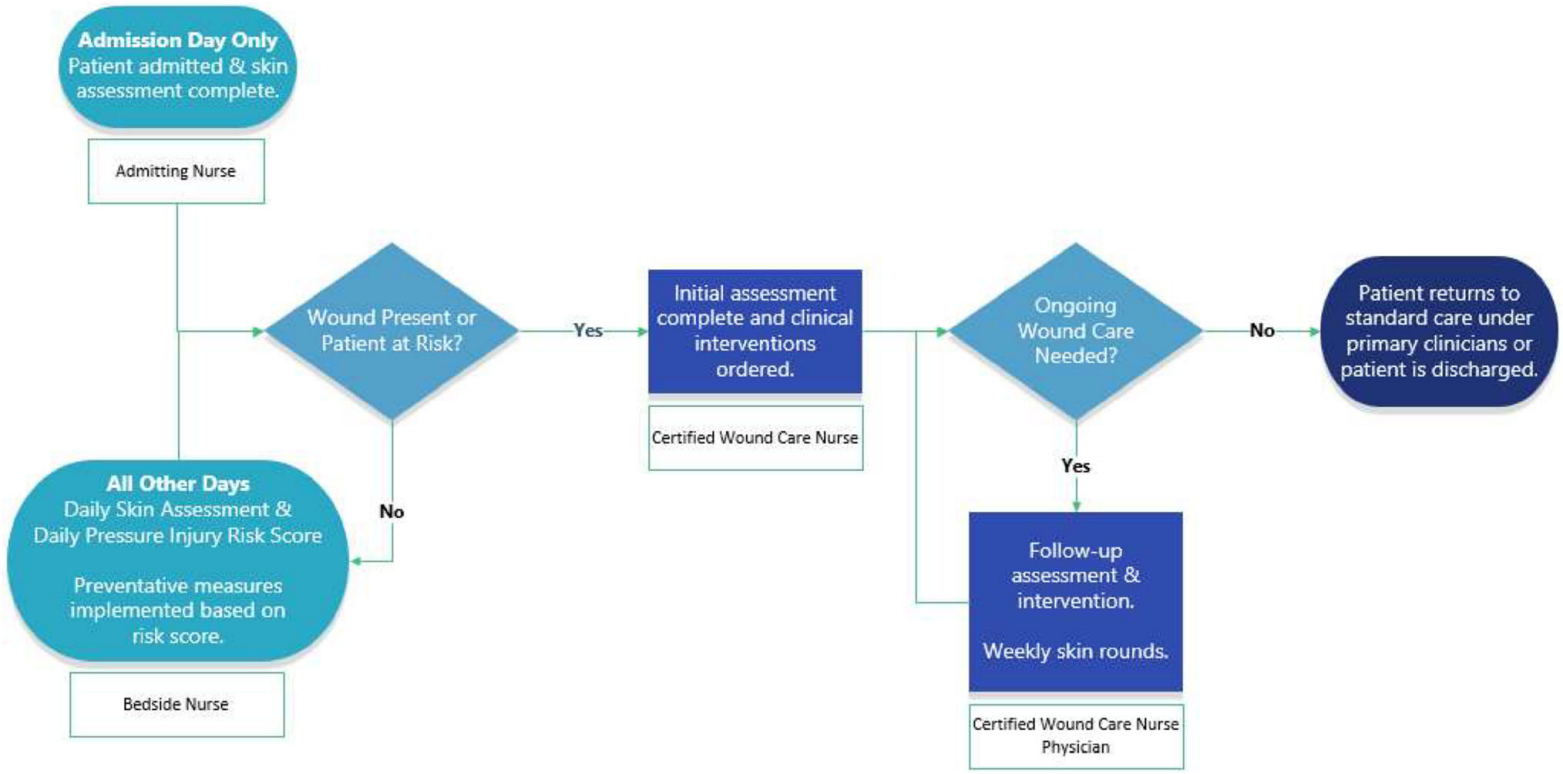
TIRR Memorial Hermann wound care protocol

The standard wound care protocol at TIRR is outlined in Figure 1 and consists of four key steps: (a) each patient is screened on admission by nursing for risk of compromised skin integrity using the Braden Scale,^22^, ^23^ and documents any existing skin breakdown in the EMR; (b) a certified WCN assesses at‐risk patients for number and severity of wounds, documenting findings and recommendations in the EMR, with these steps occurring no more than 48 hours after admission; (c) bedside nurses continue to monitor the risk of skin breakdown (Braden Scale) and evaluate skin integrity daily; (d) bedside nurses take preventive actions to support skin integrity when risk is identified. When a wound was noted, the TIRR wound treatment protocol was initiated, and individualised clinical recommendations were developed and implemented until the wound healed or the patient was discharged.

### 2.3 Data collected

Data were collected on patient demographic characteristics (age, sex, race), wound characteristics (number of wounds, wound area at baseline for superficial wounds, wound volume at baseline for non‐superficial wounds), diagnosis, medical history, complexity, and independence. Patients with a medical history of diabetes, peripheral vascular disease, peripheral arterial disease, and osteomyelitis, risk factors for slow‐healing wounds, were identified. The Case Mix Index (CMI), an indication of the complexity of an individual patient or patient population, as determined by primary diagnosis, comorbidities, age, and functional assessment upon admission, was used to compare the level of acuity between the treatment and control populations. A higher CMI number specifies a higher case complexity of the patient or patient population. Patients' level of assistance was measured using the Functional Independence Measure.

### 2.4 Outcome measures

Wound healing (“healing”) was measured as the reduction in wound surface area over a patients' inpatient stay. Progress in wound healing was examined by measuring the reduction in wound size (in surface area for superficial wounds or volume for non‐superficial wounds) at weekly intervals.

### 2.5 Statistical analyses

Two‐independent sample Student's *t*‐test was used to compare mean values of continuous patient characteristics for Treatment versus Control groups. Fisher's Exact test was used to compare the distribution of patient demographic characteristics.

The binary outcome “healing” (reduction in wound size) compared to the previous week (1 = yes, 0 = no) was modelled through a logistic mixed model with patient‐and wound‐specific random effects. Patient‐specific random effects account for the association of the outcomes in the same patient (if a patient is overall improving all his/her wounds tend to improve) and wound‐specific random effects account for the association of the outcomes at different evaluations of the same wound (if a wound is improving at an evaluation time, it tends to improve at the next time too). When fitting this model, patient‐level differences in assignment to the treatment and control groups were adjusted for, using the statistical method *Inverse Probability of Treatment Weighting* (IPTW).^24^, ^25^ The IPTW method weights each observation by the inverse of the estimated probability of assignment to the treatment group (for treatment group observations) or the control group (for control group observations). These weights are used in the estimation of model parameters. To compute the weights, the probability of being assigned to the treatment group (usually referred to as propensity score) was estimated using an ancillary logistic regression model with a binary outcome whether the patient was assigned to the treatment group or not (1 = treatment, 0 = control). The explanatory variables used for this ancillary model included demographics variables, BMI, past and present medical conditions, Functional Independence Measure (FIM) scores, wound information, and malnutrition status. The FIM is an 18‐item instrument measuring a person's level of disability in terms of burden of care, with higher levels associated with greater levels of disability.^26^

To reduce between‐group differences in the length of inpatient rehabilitation stays (a proxy for unobserved patient severity of illness), a subgroup of patients who were in inpatient rehabilitation for at least 14 days was analysed. In this more limited patient subgroup, individuals in the Control and Treatment groups were of similar age and race and had similar distributions of primary diagnosis. The outcome model (healing compared to the previous week) was estimated on the subgroup of patients with complete data in patient age, sex, race, number of wounds, log cumulative wound area at baseline (for superficial wounds), log cumulative wound volume at baseline (for non‐superficial wounds), FIM,^27^ medical history, treatment indicator, week of rehab stay indicator, and treatment‐week of rehab stay interactions. The unit of observation for this model was a patient‐week pair (N = 672). Where associations were significant, the explanatory variable was considered predictive of the outcome. Adjusted odds ratios and 95% confidence interval (CI) were estimated from this logistic regression model. Explanatory variables were considered significant at *P* < .05.

## 3 RESULTS

In the full sample, there were 227 control patients with 420 wounds and 114 treatment patients with 322 wounds. There were no statistical differences by group (Control vs Treatment) in mean age, the proportion of male/female participants, or proportion by race. Patients who received WS‐ONS had (a) higher levels of disability, as indicated by the FIM score, (b) greater severity of illness, as indicated by the CMI, (c) more and larger wounds (area of wound and number of wounds), and (d) wide variation in the number of days they were prescribed WS‐ONS (Tables 1 and 2).

**TABLE 1:** Patient characteristics ‐ full sample

	Control group	Treatment group	Group comparison test result
Patient age^a^	52.7 (19.3)	53.3 (17.9)	*P* = .79
Patient sex^b^			
Male	159 (70.0%)	90 (78.9%)	*P* = .09
Female	68 (30.0%)	24 (21.1%)	
Patient race^b^			
Black or African American	40 (17.6%)	30 (26.3%)	*P* = .39
White	115 (50.7%)	49 (43.0%)	
Asian/Not reported/Not Listed/Other	72 (31.7%)	35 (30.7%)	
Patient primary diagnosis^b^			
Brain injury	55 (24.2%)	25 (21.9%)	*P* = .56
Stroke	44 (19.4%)	16 (14.0%)	
Spinal cord injury	77 (33.9%)	47 (41.2%)	
Trauma	28 (12.3%)	16 (14.0%)	
Neurological	14 (6.2%)	4 (3.5%)	
Amputee	9 (4.0%)	6 (5.3%)	
Case mix index^a^	1.8 (0.64)	2.0 (0.67)	*P* = .01
Functional independence at admission^a^	42.8 (18.7)	38.6 (16.3)	*P* = .048
Days received WS‐ONS^a^	—	20.2 (19.7)	

^a^ Mean (SD) presented with *t*‐test comparison of group means.

^b^ Frequency count (percent of group) presented with Fisher's exact test.

**TABLE 2:** Characteristics of patient wounds ‐ full sample

	Control group	Treatment group	Group comparison test result
Number of wounds^a^	1.9 (1.2)	2.8 (1.5)	*P* < .01
Type of wounds^b^			
Burn	1 (0.2%)	6 (1.9%)	*P* < .01
Diabetic ulcer	2 (0.5%)	1 (0.35%)	
Pressure injury	135 (32.1%)	168 (52.2%)	
Surgical	119 (28.3%)	68 (21.1%)	
Trauma	14 (3.3%)	11 (3.4%)	
Venous stasis	5 (1.2%)	5 (1.6%)	
Other	144 (34.3%)	63 (19.6%)	
Average size (cm^2^) of wounds at admission^a^	12.5 (36.9)	18.4 (57.7)	*P* < .11

^a^ Mean (standard deviation) presented with *t*‐test comparison of group means.

^b^ Frequency count (percent of group) presented with Fisher's exact test.

In the sub‐group of patients with inpatient stay longer than 14 days and intervention with WS‐ONS for either 0 days (Control) or ≥14 days (Treatment), and complete observations in the variables for regression, individuals in the Control (N = 132 with 257 wounds) and Treatment (N = 49 with 152 wounds) groups were of similar age and race and had similar distributions by primary diagnosis (Table 3). Prescribing bias was decreased (Table 4) as there were no significant differences between Control and Treatment groups in terms of average wound size and functional independence, but significant differences remained between Control and Treatment in the number of wounds and in the CMI. Importantly, we found that patients in the treatment group had a greater reduction in wound surface area from baseline to discharge than did those in the Control group.

**TABLE 3:** Patient demographic characteristics in the sub‐group of patients with inpatient stay longer than 14 days and receiving wound‐specific oral nutritional supplementation (WS‐ONS) for either 0 days (control) or ≥ 14 days (treatment)

	Control (N = 132)	Treatment (N = 49)	Group comparison test result
Age, years^a^	50.7 (19.8)	50.0 (17.9)	*P* = .82
Sex^b^			
Male	92 (69.7%)	44 (89.8%)	*P* < .01
Female	40 (30.3%)	5 (10.2%)	
Race^b^			
Black or African American	23 (17.4%)	12 (24.5%)	*P* = .49
White	66 (50.0%)	21 (42.9%)	
Asian/Not reported/Not listed/Other	43 (32.6%)	16 (32.7%)	
Diagnosis^b^			
Brain injury diagnosis	34 (25.8%)	12 (24.5%)	*P* = .46
Stroke	24 (18.2%)	4 (8.2%)	
Spinal cord injury	54 (40.9%)	22 (44.9%)	
Trauma	8 (6.1%)	6 (12.2%)	
Neuro	6 (4.6%)	2 (4.1%)	
Amputee	6 (4.6%)	3 (6.1%)	

^a^ Mean (SD) presented with *t*‐test comparison of group means.

^b^ Frequency count (percent of group) presented with Fisher's exact test.

**TABLE 4:** Clinical characteristics in sub‐group of patients with inpatient stay >14 days and had either 0 days (control) or ≥ 14 days (treatment) on wound‐specific oral nutritional supplementation (WS‐ONS)

	Control patients = 132, wounds = 257	Treatment patients = 49, wounds = 152	Group comparison test result
Number of wounds at baseline^a^	1.9 (1.3)	3.1 (1.5)	*P* < .01
Wound area at baseline, cm^2^ ^a^	13.2 (38.9)	22.7 (78.4)	*P* = .16
Functional Independence Measure at baseline^a^	40.1 (16.6)	40.1 (13.1)	*P* = .10
Case Mix Index at baseline^a^	2.0 (0.6)	2.3 (0.7)	*P* < .01
Percent change wound area at discharge^a^	−33.8% (1.6)	−61.8% (0.5)	*P* = .01

^a^ Mean (SD) presented with *t*‐test comparison of group means.

**FIGURE 2:**
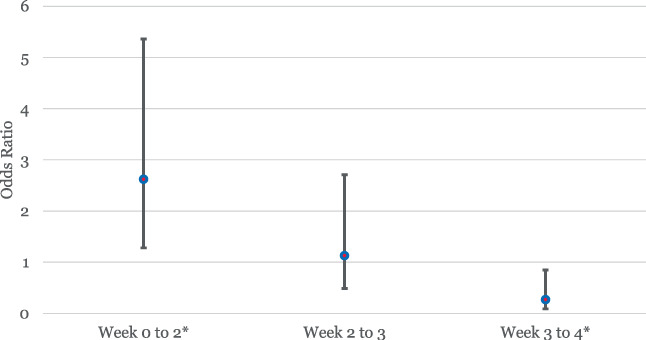
Odds of wound improvement between treatment group vs control group over time odds ratio point estimates and 95% confidence intervals(*P < .05)

The regression results of the subgroup analysis are summarised in Table 5. Positive coefficient estimates (and odds ratios greater than 1) indicate the factor was associated with an increased likelihood of wound healing from week to week; negative coefficient estimates (and odds ratios less than 1) indicate the variable was associated with a decreased likelihood of wound healing from week to week. Patients receiving WS‐ONS were more likely to exhibit a reduction in wound size in any given week, indicated by the large and significant “Treatment group” variable. However, the interactions of treatment and the week of rehab stay variables indicate that the likelihood of Treatment group patients showing greater improvement than the Control group patients diminishes over time. From baseline/admission to week 2, the odds of wound healing for patients in the treatment group were 2.62 (95% CI: 1.28, 5.36) times the odds of patients in the control group. From week 2 to week 3, these odds reduced to 1.13 (95% CI: 0.48, 2.71) and from week 3 to week 4 was 0.27 (95% CI: 0.09, 0.85), though the second odds ratio is not statistically significant (see Figure 2). Note that the number of inpatients declines over time from N = 181 for weeks 0–2 to N = 92 in weeks 2–3, and N = 54 in weeks 3–4 as patients are discharged.

**TABLE 5:** Regression of wound healing (reduction in size) on treatment and other control variables

Independent variables	Estimate (std err)	Odds ratio	*P*‐value
Treatment group	0.96 (0.37)	2.63	<.01
Week 3 of rehab stay	0.86 (0.28)	2.37	<.01
Week 4 of rehab stay	1.85 0.44	6.37	<.01
Treatment group * week 3 of rehab stay	−0.84 (0.38)	0.43	.03
Treatment group * week 4 of rehab stay	−2.25 (0.52)	0.11	<.01
Total wound area (cm^2^) baseline	−0.13 (0.07)	0.88	.06
Total wound volume (cm^3^) baseline	0.04 (0.05)	1.05	.35
Age	0.17 (0.18)	1.18	.35
Sex	0.78 (0.44)	2.18	.08
Race – Black	0.64 (0.42)	1.90	.12
Race – Other	0.22 (0.38)	1.24	.57
Number wounds	−0.33 (0.14)	0.72	.02
Medical history of diabetes, peripheral vascular disease, osteomyelitis	−1.10 (0.37)	0.33	<.01

*Note*: The main effect of treatment in Table 5 corresponded to the treatment effect from baseline/admission to week 2. Total wound area and volume at baseline were log‐transformed. Reference group for Sex and Race are Male and White respectively.

## 4 DISCUSSION

**FIGURE 3:**
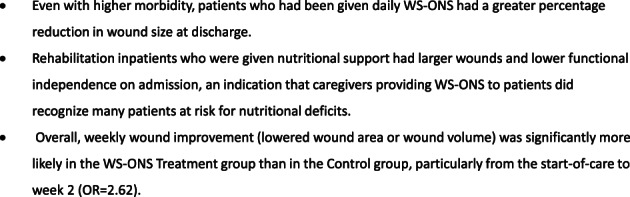
Summary of findings in our retrospective review of wound healing in a rehabilitation care site

Inpatients with the largest wounds and lowest functional independence on admission were most likely to be given WS‐ONS. Analysis in Table 4 shows wound area was significantly lower at discharge among patients who were given specialised nutritional support. Regression findings (Table 5) indicated that patients who received WS‐ONS exhibited wound healing earlier in their inpatient stay, suggesting that WS‐ONS accelerated the healing process. Week‐to‐week improvement in wound size was more likely in patients who received WS‐ONS than in those who did not (Figure 3).

### 4.1 Why it is important to solve the problem of hard‐to‐heal wounds?

Chronic wounds are common among older adults, and wound care is costly in both human and financial terms. The overall prevalence of chronic wounds was reported as 1% to 2% of the general population in developed countries.^28^, ^29^ From the US Medicare Beneficiary database, 15% of older adults experienced at least one wound or wound‐related infection, especially surgical and diabetic wounds.^4^ In a study by Deufert et al, adults receiving wound care while living at home or in a nursing home in Germany found that 48.3% of study participants had a wound duration longer than 12 months.^30^ In an Australian study of older adults (average 63,6 years) performing wound self‐care, Knapp et al found an average wound duration was 109 weeks.^31^ Study participants experienced the reduced health‐related quality of life, including negative effects on functional, psychological, social, and professional capacity.^30^, ^31^ In terms of financial costs, the Australian researchers estimated participants' out‐of‐pocket costs for wound care totaled about 10% of disposable income.^31^ Considering hospital, post‐acute, and community settings, US Medicare costs for wounds ranged from $28 to $97 billion.^4^

### 4.2 Perspectives for findings

In this analysis, we included patients with a variety of wounds in an inpatient setting for rehabilitation care, a level of care appropriate for individuals who need advanced wound care but not hospitalisation. We did not limit our study to a specific wound type. By contrast, previous research on WS‐ONS focused on its role in treating specific wound types: Ogura on pressure injuries,^16^ Armstrong on diabetic foot ulcers,^13^ Tatti and Barber on diabetic foot ulcers.^32^ By extending findings to wounds in general, we anticipate that nutritional supplementation can be applied and tested more broadly to healing different types of wounds such as surgical wounds,^33^ burns, and traumatic injuries,^19^ and oral mucosal injuries due to the effects of chemotherapy or radiation therapy.^34^, ^35^

### 4.3 How are wound‐specific ONS formulated to promote wound healing?

Nutrient deficits are known to impair wound healing, and medical nutrition therapy is a way to improve the healing of wounds. Nutrients that are vital to wound healing are (a) adequate energy by intake of carbohydrates and fats, (b) sufficient protein to promote healing processes, and (c) certain amino acids that are conditionally essential (arginine and glutamine).^17^ HMB, a metabolite of the amino acid leucine, stimulates protein synthesis pathways and reduces protein degradation,^18^ thus supporting wound healing processes. HMB in nutritional formulations has been reported to promote the healing of burns,^19^ pressure ulcers,^16^ and diabetic foot ulcers.^13^, ^32^ The fibrous protein collagen, which makes up 25% of total body protein and 70% to 80% of skin protein, is also important to wound healing.^36^ The wound‐specialised nutritional supplement used in this study is a formulation with arginine, glutamine, HMB, collagen, and micronutrients (vitamins C, E, B12, and zinc).^37^

### 4.4 Limitations of this study

Although the treatment and control groups had similar demographic characteristics, significant differences between the two groups existed in the number and size of wounds, as well as their level of function and medical complexity. In this retrospective study, patients who received WS‐ONS as interventional nutrition had more and larger wounds, indicating a prescribing bias in the data. Although inverse probability weighting was used to control for such bias, this technique assumes that there are no unmeasured confounders, that in our context means that the only factors that affect the administration or not of Juven to a patient are the predictors of the ancillary logistic model used to compute the propensity scores. There was also significant variability in the amount of WS‐ONS given to patients. The subgroup analysis focused on patients who received at least 14 days of WS‐ONS, the recommended minimum amount to see positive results. However, even within this sub‐group, there was variability in the number of days WS‐ONS treatment patients received, potentially affecting the impact of the treatment.

## 5 CONCLUSION

In an acute inpatient rehabilitation setting that provided wound care, patients who received daily WS‐ONS experienced significant wound healing within 2 weeks, which was faster than for patients on standard food only. Week‐to‐week improvement in wound size was about 2 times more likely in patients who received WS‐ONS than in those who did not. The wound‐specialised nutrition supplements contained the anabolic amino acid metabolite HMB along with amino acids arginine and glutamine and the protein collagen, all known to play important roles in skin and connective tissue integrity. Indeed, recent guidance from the American Limb Preservation Society notes that therapeutic nutrition powders, such as the one used in this study, can support wound healing by enhancing collagen production and helping to replenish critical nutrients needed for wound healing.^38^ Taken together, our findings support the benefits of wound‐specific ONS in improving wound healing for patients with pre‐existing wounds.

## FUNDING INFORMATION

Funding for this study was provided by Abbott.

## CONFLICT OF INTEREST

Rya Clark is an employee of TIRR Memorial Hermann Hospital and Research Center and a Principal Investigator of research funded by Abbott. Argyrios Stampas reports no conflict of interest. Dehuti Pandya is also an employee of TIRR Memorial Hermann Hospital and Research Center and is an investigator involved in research funded by Abbott. Kirk W. Kerr, Suela Sulo, and Jeffrey Nelson are Abbott employees and stockholders.

## Data Availability

The data that support the findings of this study are available on request from the corresponding author. The data are not publicly available due to privacy or ethical restrictions.

## References

[1] Olsson M , Friman A . Quality of life of patients with hard‐to‐heal leg ulcers: a review of nursing documentation. Br J Commun Nurs. 2020;25:S13‐S19. doi:10.12968/bjcn.2020.25.Sup12.S12 33300847

[2] Jeffcoate WJ , Vileikyte L , Boyko EJ , Armstrong DG , AJM B . Current challenges and opportunities in the prevention and management of diabetic foot ulcers. Diab Care. 2018;41:645‐652. doi:10.2337/dc17-1836 29559450

[3] Fife CE , Carter MJ . Wound care outcomes and associated cost among patients treated in US outpatient wound centers: data from the US Wound Registry. Wounds. 2012;24:10‐17. 25875947

[4] Nussbaum SR , Carter MJ , Fife CE , et al. An economic evaluation of the impact, cost, and Medicare policy implications of chronic nonhealing wounds. Value Health. 2018;21:27‐32. doi:10.1016/j.jval.2017.07.007 29304937

[5] Alam W , Hasson J , Reed M . Clinical approach to chronic wound management in older adults. J Am Geriatr Soc. 2021;69:2327‐2334. doi:10.1111/jgs.17177 34002364

[6] Jaul E , Barron J , Rosenzweig JP , Menczel J . An overview of co‐morbidities and the development of pressure ulcers among older adults. BMC Geriatr. 2018;18:305. doi:10.1186/s12877-018-0997-7 30537947 PMC6290523

[7] Lindgren M , Unosson M , Fredrikson M , Ek AC . Immobility–a major risk factor for development of pressure ulcers among adult hospitalized patients: a prospective study. Scand J Caring Sci. 2004;18:57‐64. doi:10.1046/j.0283-9318.2003.00250.x 15005664

[8] Makrantonaki E , Wlaschek M , Scharffetter‐Kochanek K . Pathogenesis of wound healing disorders in the elderly. J Dtsch Dermatol Ges. 2017;15:255‐275. doi:10.1111/ddg.13199 28252848

[9] Frykberg RG , Banks J . Challenges in the treatment of chronic wounds. Adv Wound Care. 2015;4:560‐582. doi:10.1089/wound.2015.0635 PMC4528992 26339534

[10] Palmieri B , Vadala M , Laurino C . Nutrition in wound healing: investigation of the molecular mechanisms, a narrative review. J Wound Care. 2019, 693;28:683. doi:10.12968/jowc.2019.28.10.683 31600106

[11] Saghaleini SH , Dehghan K , Shadvar K , et al. Pressure ulcer and nutrition. Indian J Crit Care Med. 2018;22(4);283‐289. doi:10.4103/ijccm.IJCCM_277_17 29743767 PMC5930532

[12] Stechmiller JK . Understanding the role of nutrition and wound healing. Nutr Clin Pract. 2010;25:61‐68. doi:10.1177/0884533609358997 20130158

[13] Armstrong DG , Hanft JR , Driver VR , et al. Effect of oral nutritional supplementation on wound healing in diabetic foot ulcers: a prospective randomized controlled trial. Diabet Med. 2014;31:1069‐1077. doi:10.1111/dme.12509 24867069 PMC4232867

[14] Basiri R , Spicer MT , Levenson CW , et al. Nutritional supplementation concurrent with nutrition education accelerates the wound healing process in patients with diabetic foot ulcers. Biomedicines. 2020;8(8):263. doi:10.3390/biomedicines8080263 PMC7460445 32756299

[15] Munoz N , Posthauer ME , Cereda E , Schols J , Haesler E . The role of nutrition for pressure injury prevention and healing: the 2019 International Clinical Practice Guideline Recommendations. Adv Skin Wound Care. 2020;33:123‐136. doi:10.1097/01.ASW.0000653144.90739.ad 32058438

[16] Ogura Y , Yuki N , Sukegane A , et al. Treatment of pressure ulcers in patients with declining renal function using arginine, glutamine and ss‐hydroxy‐ss‐methylbutyrate. J Wound Care. 2015;24:478‐482. doi:10.12968/jowc.2015.24.10.478 26488739

[17] Ghaly P , Iliopoulos J , Ahmad M . The role of nutrition in wound healing: an overview. Br J Nurs. 2021;30:S38‐S42. doi:10.12968/bjon.2021.30.5.S38 33733851

[18] Holecek M . Beta‐hydroxy‐beta‐methylbutyrate supplementation and skeletal muscle in healthy and muscle‐wasting conditions. J Cachexia Sarcopenia Muscle. 2017;8:529‐541. doi:10.1002/jcsm.12208 28493406 PMC5566641

[19] Erdem D , Sozen I , Cakirca M , et al. Effect of nutritional support containing arginine, glutamine and beta‐hydroxy‐beta‐methylbutyrate on the protein balance in patients with major burns. Turk J Anaesthesiol Reanim. 2019;47:327‐333. doi:10.5152/TJAR.2019.40327 31380514 PMC6645837

[20] Guest JF , Ayoub N , McIlwraith T , et al. Health economic burden that different wound types impose on the UK's National Health Service. Int Wound J. 2017;14:322‐330. doi:10.1111/iwj.12603 27229943 PMC7950097

[21] Dorner B , Posthauer ME , Thomas D , National Pressure Ulcer Advisory P . The role of nutrition in pressure ulcer prevention and treatment: National Pressure Ulcer Advisory Panel white paper. Adv Skin Wound Care. 2009;22:212‐221. doi:10.1097/01.ASW.0000350838.11854.0a 19521288

[22] Braden BJ , Bergstrom N . Predictive validity of the Braden scale for pressure sore risk in a nursing home population. Res Nurs Health. 1994;17:459‐470. doi:10.1002/nur.4770170609 7972924

[23] Huang C , Ma Y , Wang C , et al. Predictive validity of the Braden scale for pressure injury risk assessment in adults: a systematic review and meta‐analysis. Nurs Open. 2021;8:2194‐2207. doi:10.1002/nop2.792 33630407 PMC8363405

[24] Williamson EJ , Forbes A , White IR . Variance reduction in randomised trials by inverse probability weighting using the propensity score. Stat Med. 2014;33(5):721‐737. 24114884 10.1002/sim.5991 PMC4285308

[25] Chesnaye NC , Stel VS , Tripepi G , et al. An introduction to inverse probability of treatment weighting in observational research. Clin Kidney J. 2022;15(1):14‐20. 35035932 10.1093/ckj/sfab158 PMC8757413

[26] Dodds TA , Martin DP , Stolov WC , Deyo RA . A validation of the functional independence measurement and its performance among rehabilitation inpatients. Arch Phys Med Rehabil. 1993;74:531‐536. doi:10.1016/0003-9993(93)90119-u 8489365

[27] Granger CV , Hamilton BB , Linacre JM , Heinemann AW , Wright BD . Performance profiles of the functional independence measure. Am J Phys Med Rehabil. 1993;72:84‐89. doi:10.1097/00002060-199304000-00005 8476548

[28] Guest JF , Ayoub N , McIlwraith T , et al. Health economic burden that wounds impose on the National Health Service in the UK. BMJ Open. 2015;5:e009283. doi:10.1136/bmjopen-2015-009283 PMC4679939 26644123

[29] Heyer K , Herberger K , Protz K , Glaeske G , Augustin M . Epidemiology of chronic wounds in Germany: analysis of statutory health insurance data. Wound Repair Regen. 2016;24:434‐442. doi:10.1111/wrr.12387 26609788

[30] Deufert D , Graml R . Disease‐specific, health‐related quality of life (HRQoL) of people with chronic wounds—a descriptive cross‐sectional study using the wound‐QoL. Wound Med. 2017;16:29‐33.

[31] Kapp S , Santamaria N . The financial and quality‐of‐life cost to patients living with a chronic wound in the community. Int Wound J. 2017;14:1108‐1119. doi:10.1111/iwj.12767 28635188 PMC7949507

[32] Tatti P , Barber A . The use of a specialized nutritional supplement for diabetic foot ulcers reduces the use of antibiotics. J Endocrinol Metab. 2012;2:26‐31.

[33] Nishizaki K , Ikegami H , Tanaka Y , Imai R , Matsumura H . Effects of supplementation with a combination of beta‐hydroxy‐beta‐methyl butyrate, L‐arginine, and L‐glutamine on postoperative recovery of quadriceps muscle strength after total knee arthroplasty. Asia Pac J Clin Nutr. 2015;24:412‐420. doi:10.6133/apjcn.2015.24.3.01 26420181

[34] Naganuma A , Hoshino T , Ohno N , et al. Beta‐Hydroxy‐beta‐methyl Butyrate/L‐Arginine/L‐Glutamine supplementation for preventing hand‐foot skin reaction in sorafenib for advanced hepatocellular carcinoma. In Vivo. 2019;33:155‐161. doi:10.21873/invivo.11452 30587616 PMC6364076

[35] Yuce Sari S , Yazici G , Yuce D , et al. The effect of glutamine and arginine‐enriched nutritional support on quality of life in head and neck cancer patients treated with IMRT. Clin Nutr ESPEN. 2016;16:30‐35. doi:10.1016/j.clnesp.2016.08.003 28531452

[36] Riekki R , Parikka M , Jukkola A , et al. Increased expression of collagen types I and III in human skin as a consequence of radiotherapy. Arch Dermatol Res. 2002;294:178‐184. doi:10.1007/s00403-002-0306-2 12111348

[37] Abbott Laboratories . Juven: Healthcare Professionals. Columbus, OH: Abbott Laboratories. http://juven.com/hcp (Accessed October 7, 2021).

[38] Armstrong DA , Mills JL , Molina M , et al. Nutrition interventions in adults with diabetic foot ulcers: expert consensus and guidance. Ame Limb Preserv Soc. 2022; Accessed April 11, 2022 at http://eguideline.guidelinecentral.com/i/1428995-nutrition-in-dfu-guidelines-advisory-pocket-guide/19

